# An optimized method for high quality DNA extraction from microalga *Prototheca wickerhamii* for genome sequencing

**DOI:** 10.1186/s13007-017-0228-9

**Published:** 2017-10-03

**Authors:** Tomasz Jagielski, Jan Gawor, Zofia Bakuła, Karolina Zuchniewicz, Iwona Żak, Robert Gromadka

**Affiliations:** 1 Department of Applied Microbiology, Institute of Microbiology, Faculty of Biology, University of Warsaw, I. Miecznikowa 1, 02-096 Warsaw, Poland; 2 DNA Sequencing and Oligonucleotides Synthesis Laboratory at the Institute of Biochemistry and Biophysics, Polish Academy of Sciences, A. Pawińskiego 5a, 02-106 Warsaw, Poland; 3 Department of Clinical Microbiology, Children’s University Hospital of Cracow, Kraków, Poland

**Keywords:** DNA extraction, DNA isolation, Genome sequencing, *Prototheca*

## Abstract

**Background:**

The complex cell wall structure of algae often precludes efficient extraction of their genetic material. The purpose of this study was to design a next-generation sequencing-suitable DNA isolation method for unicellular, achlorophyllous, yeast-like microalgae of the genus *Prototheca*, the only known plant pathogens of both humans and animals. The effectiveness of the newly proposed scheme was compared with five other, previously described methods, commonly used for DNA isolation from plants and/or yeasts, available either as laboratory-developed, in-house assays, based on liquid nitrogen grinding or different enzymatic digestion, or as commercially manufactured kits.

**Results:**

All five, previously described, isolation assays yielded DNA concentrations lower than those obtained with the new method, averaging 16.15 ± 25.39 vs 74.2 ± 0.56 ng/µL, respectively. The new method was also superior in terms of DNA purity, as measured by A260/A280 (−0.41 ± 4.26 vs 2.02 ± 0.03), and A260/A230 (1.20 ± 1.12 vs 1.97 ± 0.07) ratios. Only the liquid nitrogen-based method yielded DNA of comparable quantity (60.96 ± 0.16 ng/µL) and quality (A260/A280 = 2.08 ± 0.02; A260/A230 = 2.23 ± 0.26). Still, the new method showed higher integrity, which was best illustrated upon electrophoretic analysis. Genomic DNA of *Prototheca wickerhamii* POL-1 strain isolated with the protocol herein proposed was successfully sequenced on the Illumina MiSeq platform.

**Conclusions:**

A new method for DNA isolation from *Prototheca* algae is described. The method, whose protocol involves glass beads pulverization and cesium chloride (CsCl) density gradient centrifugation, was demonstrated superior over the other common assays in terms of DNA quantity and quality. The method is also the first to offer the possibility of preparation of DNA template suitable for whole genome sequencing of *Prototheca* spp.

## Background

The genus *Prototheca* (Trebouxiophyceae) accommodates unicellular, achlorophyllous, yeast-like microalgae, ubiquitously distributed in the environment. Although normally saprophytic, these organisms may, under certain conditions, give rise to infections in both humans and animals. The *Prototheca* algae are hence the only known plant causative agents of human and animal disease [1].

The scientific knowledge on the *Prototheca* algae is very limited. One of the major gaps has been a paucity of understanding of the pathobiology and mechanisms underlying the protothecal disease. For this to be revealed, a considerable amount of genetic data is required. Of particular importance would be those derived from the whole-genome sequencing (WGS) studies. Until now, however, no such reports have been published, a possible reason for this being the lack of a rapid and efficient method for high-quality genomic DNA extraction from *Prototheca* spp.

Most of the protocols currently used for DNA extraction from *Prototheca* algae, are essentially the same as those applied for plants and/or fungi and usually exploit a variety of physical disruption methods of cell lysis, including liquid homogenization, sonication, and grinding in liquid nitrogen [2–8]. These methods, though very useful and robust for many fungal or plant species, produce little amounts of protothecal DNA, which is often highly impure and prone to shearing. Whereas such DNA can still be used as a template for single-locus PCR amplification, and subsequent sequencing, an approach commonly employed for *Prototheca* species- (genotype-) level identification [6, 9], it is insufficient for WGS purposes.

A combination of high concentration and high purity of DNA, with no evidence of contamination from polysaccharides, proteins or RNA, with maximally reduced fraction of fragmented and chemically degraded DNA is a prerequisite for all next-generation sequencing (NGS) technologies [10, 11].

The difficulty of the DNA isolation from eukaryotic microalgae has repeatedly been reported [12–15] and this has been attributed to their unique cell wall structures, whose constituents include some highly resistant biomolecules, such as algaenans, dinosporins or silica compounds [16, 17]. In *Prototheca* species it is sporopollenin, a complex biopolymer, which chiefly renders the algae hyper-refractory to various chemical and physical treatments used to disrupt plant cell walls [18, 19].

The purpose of this study was to design a next-generation sequencing-suitable DNA isolation method for *Prototheca* microalgae. The effectiveness of the newly proposed scheme was compared with five other, previously described methods, commonly used for DNA isolation from plants and/or yeasts available either as laboratory-developed, in-house assays or as commercially manufactured kits.

## Methods

### Strain

*Prototheca wickerhamii* POL-1 strain, originally isolated at the Department of Clinical Microbiology, Children’s University Hospital of Kraków, from the cerebrospinal fluid of a 6-month child with the signs of neuroinfection, which was proved to be the first case of human protothecosis in Poland [20], was used in this study.

### Cell growth condition

Cells of *P. wickerhamii* POL-1 strain were picked from a single colony on Yeast-Peptone-Dextrose (YPD) agar (Sigma, Saint Louis, USA) and grown in a 100 mL volume of YPD broth for 72 h at 37 °C with shaking (200 rpm) until the optical density at A600 reached ca. 5.0 (ca. 6.5 × 10^5^ CFU).

### Genomic DNA extraction protocols

Six DNA extraction protocols were evaluated in this study. DNA was isolated in triplicate with each protocol. The first experimental steps were always the same and aimed at separating the algal cells from the medium. Shortly, a total volume of 10 mL of liquid culture was centrifuged (10 min, 5000 rpm, RT), and the obtained pellet was suspended in 1 mL of Tris–EDTA (TE; 10 mM Tris–HCl pH 7.6, 0.1 mM EDTA). This was repeated twice to ensure complete removal of growth medium. At the end, washed cell pellet was suspended in a proper buffer, depending on the chosen protocol’s specification.

### Extraction with grinding in liquid nitrogen (LN)

First extraction method was performed as described by van Burik et al. [7]. Briefly, the algal cells, suspended in TE buffer, were ground to a fine powder, by using an autoclaved, pre-chilled mortar and pestle. The powdered sample was resuspended in 600 µL of cetyltrimethylammonium bromide (CTAB) extraction buffer (1% CTAB (Sigma, Saint Louis, USA), 1.4 M NaCl, 100 mM Tris pH 8.0, 20 mM EDTA), transferred to a 2-mL microcentrifuge tube and incubated on ice for 1 h. DNA was further extracted using phenol–chloroform–isoamyl alcohol (Phe/Chl/IAA, 25:24:1) (Sigma, Saint Louis, USA) followed by isopropanol DNA precipitation. The obtained pellet was resuspended in 100 µL TE buffer with RNAse A (50 µg/mL, Sigma, Saint Louis, USA), and after centrifugation (5 min, 14,000 rpm, RT), the cellular debris was removed, while clear supernatant was transferred to a new tube.

### Extraction with grinding in CTAB (C)

Second extraction method was that described by Doyle [8]. The algal pellet was ground in pre-warmed (60 °C) CTAB isolation buffer (2% CTAB (Sigma, Saint Louis, USA), 1.4 M NaCl, 100 mM Tris pH 8.0, 20 mM EDTA). The mixture was then transferred to a 2-mL microcentrifuge tube and incubated at 60 °C for 1 h. DNA was extracted once with chloroform-isoamyl alcohol (Chl/IAA, 24:1) (Sigma, Saint Louis, USA) and precipitated with two volumes of isopropanol. The obtained pellet was washed with 70% EtOH, dried, and dissolved in 100 µL TE buffer with RNAse A (50 µg/mL, Sigma, Saint Louis, USA).

### Extraction by enzyme cocktail (EC)

Another isolation method was based on a CTAB protocol, previously used for DNA isolation from *Chlorella variabilis* NC64A [21], with an additional cell wall digestion step with polysaccharide-degrading enzymes, suitable for *Prototheca* spp. [22]. Here, the algal cells were resuspended in 900 µL of TE buffer and then 100 µL of enzyme cocktail containing lyticase (100 µg/mL, Sigma, Saint Louis, USA), cellulase Onozuka RS (1 mg/mL, Yakult Pharmaceutical Industry, Tokyo, Japan), pectolyase (1 mg/mL, Sigma, Saint Louis, USA), and pectinase (1 mg/mL, Sigma, Saint Louis, USA) was added. The cell suspension was incubated at 37 °C for 3 h. Cell lysis was continued by addition of 100 µL of 10% SDS and Proteinase K (10 µg/mL, Sigma, Saint Louis, USA), followed by incubation at 56 °C for 1 h. After cell lysis, 200 µL of 5 M NaCl was added to the sample and mixed thoroughly. Afterwards, 160 µL of CTAB, prewarmed to 65 °C, was added, followed by 10 min of incubation at 65 °C. The lysate was then extracted four times with an equal amount of Phe/Chl/IAA (25:24:1) until the interface was clear. DNA was precipitated by addition of 0.7 volume of isopropanol and centrifugation (20 min, 14,000 rpm, RT). DNA pellet was washed once with 1 mL of 70% ethanol, air-dried, dissolved in 200 µL of TE buffer with RNase A (50 µg/mL), and incubated at 37 °C for 30 min with shaking. Samples were then spun in a microcentrifuge (5 min, 14,000 rpm, RT), and clear supernatant was transferred to a new tube.

### Extraction with commercial kits (K1 and K2)

Two commercially available kits, designed for rapid purification of genomic DNA, based on specific buffer formulations and DNA-binding silica membrane columns, were tested, namely GeneMATRIX Bacterial & Yeast Genomic DNA Purification Kit (EurX^®^, Gdańsk, Poland), in combination with lyticase (100 μg/mL) and β-mercaptoethanol (β-ME, 1 μL/mL) (Sigma, Saint Louis, USA) (K1) and GeneMATRIX Plant & Fungi DNA Purification Kit (EurX^®^, Gdańsk, Poland) (K2). When using both kits, all steps were performed strictly in accordance with instructions provided by the manufacturer.

### Extraction with glass beads: a new protocol (N)

The cell pellet from culture medium was suspended in 750 µL of an extraction buffer (2% Triton-X100, 1% SDS, 100 mM NaCl, 10 mM Tris–HCl pH 8.0, 1 mM EDTA) [7], and the suspension was transferred into 2-mL microcentrifuge tube. Lysis of the algae was achieved by pulverization with 0.4–0.6-mm diameter glass beads (Sartorius AG, Göttingen, Germany), in a 1:1 ratio, in a tissue lyser (TissueLyser II; Qiagen, Hilden, Germany) at 20 Hz for 15 min. The sample was then transferred into a 5-mL microcentrifuge tube. The glass beads were washed two times with 500 µL of an extraction buffer, and the washes were pooled and added to the homogenate, so that its final volume was ca. 2.5 mL. Cell lysis was continued by adding Proteinase K (160 µg/mL) and incubation at 56 °C for 1 h. After that time, 500 µL of 5 M NaCl was added and mixed thoroughly. Next, 400 µL of CTAB, prewarmed to 65 °C, was added followed by 10 min of incubation at 65 °C. The lysate was then extracted with an equal volume of Phe/Chl/IAA (24:24:1), repeated four times until no protein interphase could be seen. DNA was precipitated with 0.7 volume of isopropanol, followed by centrifugation (20 min, 14,000 rpm, RT), and washing with 1 mL of 70% ethanol. The resulting pellet was air-dried and resuspended in 200 µL of TE buffer with RNAse A (50 µg/mL). Following an incubation at 37 °C with gently shaking for 30 min, DNA was centrifuged again (5 min, 14,000 rpm, RT), and the clear supernatant was collected in a new tube.

### Optional sample clean-up prior to DNA concentration measurements

To all the in-house methods (LN, EC, N) an extra step was added to remove any residual ribonucleotides, proteins, and other possible contaminants which might interfere with sample quality. This was achieved by adding to the DNA precipitate, after RNAse treatment, an equal volume of Chl/IAA (24:1) (Sigma, Saint Louis, USA). Once centrifuged (5 min, 14,000 rpm, RT), the supernatant was taken for re-precipitation of DNA with 0.7 volume of isopropanol. This was followed by centrifugation (15 min, 14,000 rpm, RT), washing with 500 µL of 70% ethanol, air-drying, and re-suspension in 100 µL of TE buffer.

### Nuclear DNA purification

DNA sample obtained with the N method was used in this procedure. To separate nuclear DNA from mitochondrial and plastid DNA, cesium chloride (CsCl) density gradient ultracentrifugation was performed, essentially as described before [23]. Shortly, DNA sample was transferred to a centrifuge tube, containing 8.6 g of CsCl and 1 mL of ethidium bromide (10 μg/mL, Sigma, Saint Louis, USA) and filled up with TE buffer to a final volume of 8 mL. After centrifugation (5 min, 10,000 rpm, RT), samples were decantated to a new tube and ultracentrifuged (48 h, 45,000 rpm, 15 °C, Beckman, Ti50 rotor, Indianapolis, USA). After the ultracentrifugation, the brighter upper band, expected to represent nuclear DNA fraction, was collected with a pipette under UV transilluminator. To remove CsCl from DNA solution a dialysis was carried out in cellulose membranes (MWCO = 140,000; Sigma Saint Louis, USA) at 4 °C for 20 h with one TE buffer change. After dialysis samples were collected and concentrated using the Amicon Ultra 0.5 mL 30 K columns in accordance to the manufacturer’s instructions (Merck Millipore, Darmstadt, Germany).

### DNA concentration and purity

For each extraction procedure, the quantity and purity of template DNA was assessed based on the absorbance readings at 230, 260, and 280 nm, and calculated 260:280 and 260:230 ratios, using a PicoDrop spectrophotometer (PicoDrop Ltd, Hinxton, UK). Concentration of the genomic DNA was estimated fluorometrically using the High Sensitivity DNA kit and Qubit 2.0 fluorometer (Thermo Fisher Scientific, Waltham, USA). Each time 1 µL (fluorescence) or 2 µL (absorbance) of DNA sample (or TE buffer as a blank solution) was used. All measurements were done in duplicate.

### DNA integrity

The integrity of genomic DNA, isolated with five tested methods, was assessed by standard electrophoresis, pulsed field gel electrophoresis (PFGE) and field-inversion gel electrophoresis (FIGE). Firstly, DNA samples were resolved electrophoretically on a 1% agarose gel. Secondly, samples whose DNA concentrations were more than 2 ng/µL (methods LN, K1, K2, N) were subjected to PFGE and FIGE analysis to visualize smaller (> 45 kb) and larger (< 45 kb) DNA fragments, respectively. Both these analyses were performed with a CHEF Mapper system (BioRad, Hercules, USA), following the manual instructions [24], on 1.0% Pulsed Field Certified Agarose gels (BioRad, Hercules, USA) in 0.5 × Tris–Borate EDTA (TBE; 40 mM Tris–HCl pH 8.3, 45 mM boric acid, 1 mM EDTA) pre-chilled to 14 °C. PFGE was run for 18 h at an angle of 120°, with an initial switching time of 0.35 s and a final switching time of 8.53 s, at 6 V/cm. For FIGE analysis, 24-h run was used with a switch time logarithmically ramping from 0.22 to 0.92 s, and with a ramp factor of 0.357 (21%). Forward and reverse voltage gradients were 9 V/cm (300 V) and 6 V/cm (200 V), respectively. Gels were stained with ethidium bromide (10 μg/mL) and visualized using UV transilluminator.

### Quality assessment of extracted DNA

To assess the quality and purity of nuclear DNA extracted with a new, optimized protocol, and to evaluate its usefulness for genome sequencing, the WGS was performed with the Illumina MiSeq platform (Illumina, San Diego, USA). This was done for two genomic DNA samples obtained by the N method with and without CsCl ultracentrifugation to ensure that this step is crucial for separation of nuclear from organellar DNA. The pair-end sequencing library construction was performed with 1 µg of post-nebulized DNA extract and the KAPA Library Preparation Kit reagents (KAPA Biosystems, Wilmington, USA), according to manufacturer’s instructions. The libraries were quality checked on an agarose (1.5%) gel, pooled and sequenced on a MiSeq instrument using the MiSeq reagent Kit v3 (600 cycle) chemistry (Illumina, San Diego, USA).

Once obtained, sequence reads were quality filtered using FastaX toolkit [25], and the remaining sequencing adaptors were removed by Cutadapt [26]. Nuclear, mitochondrial, and plastid genomes of *P. wickerhamii* POL-1 strain were entirely sequenced in the course of the Polish *P. wickerhamii* WGS project (manuscript in preparation). All the bioinformatic manipulations were done using the CLCBio Genomic Workbench NGS pipeline [27].

To estimate separation of nuclear DNA from organellar DNA, one million of Illumina sequencing paired reads (2 million of sequence reads) were randomly subsampled from *P. wickerhamii* POL-1 strain library dataset. The sequences were searched against mitochondrial and plastid genomes of *P. wickerhamii* POL-1.

The read quality distribution graph was generated using CLC Bio Genomic Workbench software (Version 9.0; Qiagen, Hilden, Germany).

## Results and discussion

### DNA concentration and quality

Basic DNA quality measures, for each extraction method tested, are listed in Table 1. Of the six methods under the study, the efficiency of three (EC, K1, and K2) was low with DNA concentrations less than 4 ng/µL, as evidenced by fluorometry. The enzymatic method (EC) performed the worst both in terms of DNA quantity (1.6 ± 0.01 ng/µL) as well as A260:280 (−7.97 ± 19.25) and A260:230 (0.15 ± 0.02) ratios. The absorbance ratios were also far from satisfactory (A260:280 = 1.32 ± 0.50 and 0.70 ± 0.29, A260:230 = 0.51 ± 0.07 and 0.53 ± 0.17 for K1 and K2, respectively) in case of DNA extraction by using silica membrane-based spin columns (K1 and K2). The low A260:280 ratios may either be due to heavy protein contamination or residual phenol associated with the extraction protocol. Whereas, the low A260:230 ratios may either indicate residual phenol or carbohydrate carryover, a problem commonly encountered upon DNA isolation from plants [28]. For samples extracted with the LN, C, and N methods, the A260:280 ratios fell within the range of 2.06–2.1, 1.81–1.87, and 1.99–2.05, respectively, whereas the A260:230 ratios were within the range of and 1.97–2.49, 2.57–2.61, and 1.92–2.05 respectively. These values are consistent with the absence of proteins and other organic contaminants.

**Table 1:** Comparison of five different DNA extraction methods for *P. wickerhamii* POL-1 strain in terms of purity and yield

Method	Absorbance		Concentration (ng/µL)
	260:280	260:230	
Liquid nitrogen (LN)	2.08 ± 0.02	2.23 ± 0.26	60.96 ± 0.16
CTAB (C)	1.84 ± 0.03	2.59 ± 0.02	12.07 ± 0.7
Enzyme cocktail (EC)	−7.97 ± 19.25	0.15 ± 0.02	1.6 ± 0.01
GeneMATRIX Bacterial & Yeast (K1)	1.32 ± 0.50	0.51 ± 0.07	2.28 ± 0
GeneMATRIX Plant & Fungi (K2)	0.70 ± 0.29	0.53 ± 0.17	3.85 ± 0.02
Newly designed protocol (N)	2.02 ± 0.03	1.97 ± 0.07	74.2 ± 0.56

Although the purity indicators of the three methods (LN, C, and N) were quite the same, the N method yielded DNA of higher concentrations (74.2 ± 0.56 vs 12.07 ± 0.7 vs 60.96 ± 0.16 ng/µL).

**Fig. 1:**
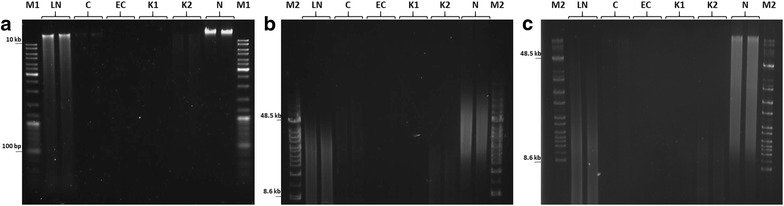
Evaluation of DNA integrity. Standard electrophoresis (**a**), PFGE (**b**), and FIGE (**c**) analysis of genomic DNA (10 µL) isolated by different methods: liquid- (LN), CTAB- (C), enzyme cocktail (EC)-based, commercial kits (K1 and K2), and glass beads pulverization-based, new protocol (N). M1, GeneRuler 1 kb DNA Ladder (Thermo Fisher Scientific, Waltham, USA), M2, CHEF DNA Size Standards – 8–48 kb (BioRad, Hercules, USA)

Standard electrophoresis of DNA extracts showed the best results for the N method, both in terms of DNA yield and integrity. Whereas methods EC, K1, K2, and C generated clearly smaller DNA amounts, method LN produced DNA less intact and more sheared (Fig. 1a).

To further inspect the integrity of DNA samples obtained with different extraction methods, PFGE and FIGE analyses were performed. PFGE analysis revealed that all the DNA templates were sheared during the extraction procedure. Yet the N method resulted in somewhat narrower fragment length distribution, with slightly greater contribution of large fragments compared to the LN method (Fig. 1b).

DNA isolation methods generating high molecular weight DNA fragments have been considered more suitable for second generation sequencing technology such as Illumina and third-generation sequencing technologies capable of producing long reads, such as Pacific Biosciences single-molecule real-time (SMRT) or Oxford Nanopore sequencing [29, 30]. Long DNA fragments are crucial for high quality libraries preparation and further efficient genome assembly using long reads [31].

Similar observations were concluded with the FIGE analysis, i.e. low DNA concentration with a length distribution weighted more towards shorter fragments (K1 and K2) and high DNA concentration with much broader fragment length distribution (LN and N) (Fig. 1c). As repeatedly argued, the higher average molecular weight of the fragments, clearly seen on electropherograms, the better is the quality of the genomic template [32, 33].

### NGS sequencing quality

**Fig. 2:**
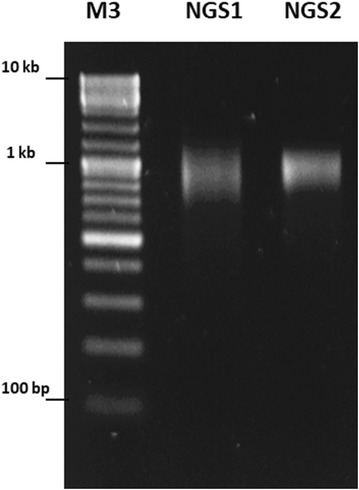
Evaluation of the Illumina sequencing library quality. Standard 1.5% agarose electrophoresis gel image of two Illumina DNA libraries, generated using DNA extracted without (NGS1) and with CsCl purification (NGS2). M3, GeneRuler Ladder Mix (Thermo Fisher Scientific, Waltham, USA)

Libraries for *P. wickerhamii* POL-1 genomic DNA extracted with a newly designed (N) protocol, with and without CsCl ultracentrifugation step, were constructed (Fig. 2) and successfully sequenced on Illumina MiSeq platform with an average insert size of 760 and 750 bp and yielding 2,342,210 and 10,173,050 reads, respectively (Table 2). The ultracentrifugation step was demonstrated advantageous for removal of the extranuclear DNA, as evidenced by reduced number of reads mapped to mitochondrial and plastid genome sequence of the *P. wickerhamii* POL-1 strain (Table 3). The number of sequences aligned to mtDNA and ptDNA after additional purification decreased from 56,536 to 2686 and from 77,327 to 4869, respectively. The overall number of reads mapped to organellar genomes decreased by nearly 18 times (from 6.69 to 0.38%) when the CsCl gradient step was applied.

**Table 2:** Summary of *P. wickerhamii* POL-1 genomic DNA sequencing results

Sample	Amount of DNA for library preparation (ng)	Sequencing reads—total	Sequencing bases—total
With CsCl	1000	2,342,210	703,254,704
Without CsCl	1000	10,173,050	3,033,748,450

**Table 3:** A total number of reads unmapped and mapped to mitochondrial and plastid genome sequence of the *P. wickerhamii* POL-1 strain

Sample	Reads count		
	Unmapped—nuclear DNA	Mapped (mtDNA + ptDNA)	Mapped %
With CsCl	1,866,137	133,863 (56,536 +77,327)	6.69
Without CsCl	1,992,445	7555 (2686 + 4869)	0.38

**Fig. 3:**
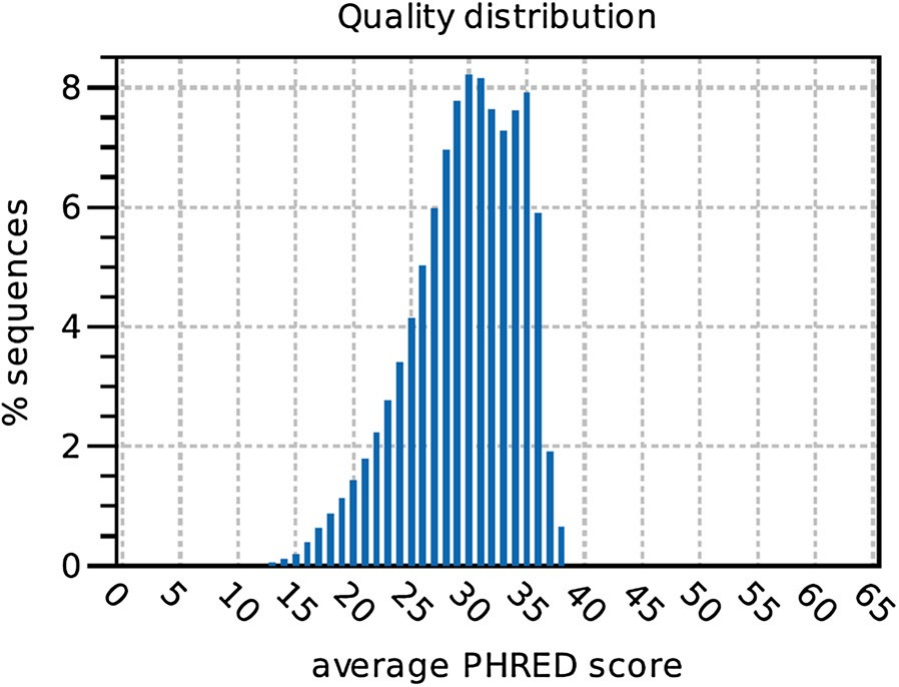
The quality of *Prototheca wickerhamii* POL-1 genomic DNA sequence. The x-axis represents the PHRED quality score and the y-axis represents the percentage of sequences with a quality score, normalized to the total number of sequences

To measure the quality of the identification of the nucleotide bases generated by sequencing, PHRED quality score was estimated for protothecal DNA samples extracted with CsCl ultracentrifugation (Fig. 3). Average PHRED score was calculated at the level of 30 which implies high confidence in the quality of DNA submitted (with base call accuracy of 99.9%) [34].

The genome of the *P. wickerhamii* POL-1 strain was entirely sequenced in the course of the Polish *P. wickerhamii* WGS project. The sequencing yielded 2860 scaffolds with the total assembly size of 29 Mbps (manuscript under preparation).

The reason for which the NGS was performed only on DNA isolated with the N method, and not with the others, including the “LN” method was not only better parameters for DNA quantity and purity (Table 1), but also its higher integrity, as assessed by electrophoretic methods, especially PFGE and standard electrophoresis. As shown in Fig. 1, the N method produced DNA template less sheared and of narrower fragment length distribution. This is of particular notice that the N method yielded more larger fragments compared with the LN method. This can be seen on inspecting Fig. 1, with a clear shift towards fragments of higher molecular weight or a height of large DNA fragments having greater intensities, respectively; the central molecular size of the fragments’ range was ca. 33.5–38.4 kb for the LN method, and ca. 48.5 kb for the N method.

Long DNA fragments are mandatory for high quality libraries preparation for NGS technologies and further efficient de novo genome assembly especially with long reads.

All this argued for the superiority of the N method over the other ones, including the LN method, and guided our decision of using this particular method (N) in further experimental steps, such as the CsCl purification, and finally sequencing.

## Conclusions

In the present study, for the first time, an efficient and reliable procedure of protothecal DNA extraction for whole-genome sequencing and large-scale genotyping studies was described. The protocol herein proposed incorporates some key technological solutions from previously described assays of plant and fungal DNA isolation, and involves a three-pronged approach for whole cell lysis, i.e. mechanical (glass bead pulverization), surfactant-based (Triton-X100, SDS, and CTAB treatment), and enzymatic (Proteinase K treatment) disruption methods. An important step in the protocol is the CsCl density gradient ultracentrifugation allowing for separation of nuclear DNA from extranuclear (organellar) DNA. To the best of authors’ knowledge, a method combining all these components has never been attempted to isolate genomic DNA from either microalgae or any other plant species.

The method here reported represents a considerable improvement over the present methods of DNA isolation from the cell-walled eukaryotes. The key advantages are a good yield and high quality (purity and integrity) of DNA, affording different molecular genotyping technologies, including NGS. Perhaps the only drawback of the method is that the procedure is quite time-consuming, with a turnaround time of 3–4 days before the specimen can achieve a ready-to-use form.

One may argue why did we not apply some other, already existing protocols of DNA extraction from microalgae. For instance, in the early 2000s, the nucleotide sequence of the *P. wickerhamii* ptDNA was determined by using, for DNA isolation, a liquid nitrogen-based method [35]. More recently, Tourasse et al. have reported the complete mitochondrial genome sequence of *Lobosphaera* (*Parietochloris*) *incisa*, an oleaginous unicellular green alga belonging to the class Trebouxiophyceae, the same that the *Prototheca* genus is affiliated with. Here, DNA was extracted following the standard CTAB DNA extraction protocol, whose original description was provided 30 years ago [36]. Both these methods, under the LN and C designations, respectively, were tested in our study. However, in terms of at least DNA concentration and DNA size distribution, which are among the key parameters for NGS, both these methods were inferior to our newly-developed N method. At this point, it is worth considering two issues. First, a method of DNA isolation, useful for some species, may not be equally or at all effective for other, even closely-related species. This may be due to even discrete differences in the cell wall composition, especially the content and/or distribution of chemically refractory compounds, such as sporopollenin, a distinctive constituent of the protothecal cell wall. Second, a method of DNA isolation, which allows for sequencing of the extrachromosomal DNA (ptDNA and/or mtDNA) may not be suitable for whole-genome sequencing [37]. It is remarkable that with a plethora of DNA extraction methods available, never has the genomic DNA, other than organellar, been sequenced in *Prototheca* spp.

Since the method here proposed is the first to offer the possibility of preparation of protothecal DNA template suitable for WGS, it paves the way for large-scale investigations into genomics and proteomics of the *Prototheca* spp., and possibly other microalgae and plants.
